# Human Papillomavirus Positive Squamous Cell Carcinoma of the Rectum

**DOI:** 10.7759/cureus.9022

**Published:** 2020-07-06

**Authors:** Shraddhadevi Makadia, Ishan Patel, Khalid Abusaada

**Affiliations:** 1 Internal Medicine, Ocala Regional Medical Center, University of Central Florida College of Medicine, Ocala, USA; 2 Internal Medicine, Jersey Shore University Medical Center, Neptune, USA

**Keywords:** squamous cell carcinoma, rectal carcinoma, human papillomavirus (hpv)

## Abstract

Squamous cell carcinoma (SCC) is most commonly seen in the esophagus and anal canal in the gastrointestinal tract. The incidence of SCC of the rectum is infrequent with no clear etiology. There have been limited reported cases of SCC of the rectum caused by human papillomavirus (HPV). Due to the rarity of carcinoma, the management of SCC of the rectum is not standardized. We report a case of a 51-year-old female with an insignificant medical history presenting with hematochezia and weight loss and was found to have HPV-positive SCC of the rectum. This case report emphasizes the importance of work-up, usefulness of HPV testing for high-risk patients, and clinical management of SCC of the rectum.

## Introduction

Squamous cell carcinoma (SCC) of the rectum is a very rare malignancy, accounting for approximately 0.10-0.25 per 1,000 colorectal neoplasms [1]. Human papillomavirus (HPV) type 16 associated SCC of the rectum is an exceedingly rare malignancy. The first reported case of SCC of the colon was in a 65-year-old man by Schmidtmann in 1919 [2]. Although the pathogenesis of SCC is not well understood, an association with HPV infection is hypothesized to play a role. The first documented SCC of the rectum with positive HPV-16 was reported in an 87-year-old man by Sotlar et al. in 2001 [3]. We present a case of a 51-year-old woman with HPV positive SCC of the rectum who presented with a two-month history of hematochezia and weight loss.

## Case presentation

**Figure 1:**
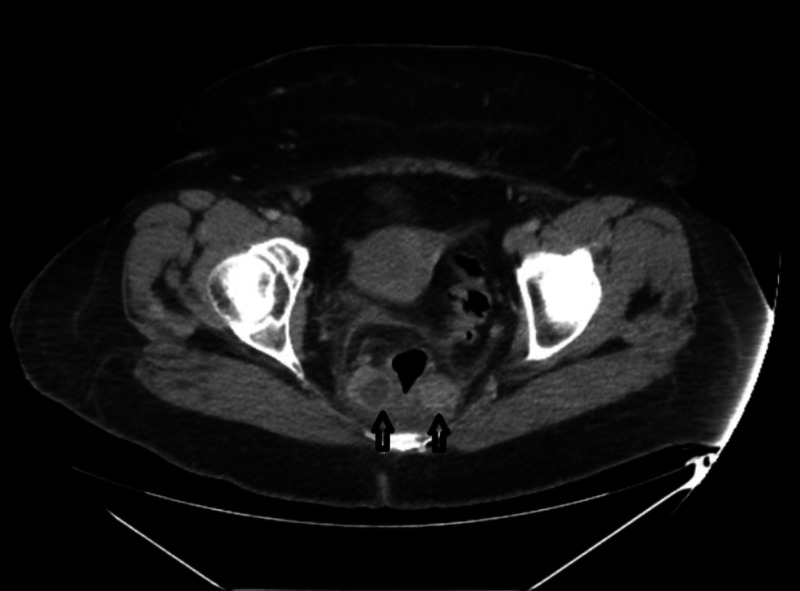
CT scan of the pelvis. Perirectal: 17-mm perirectal abscess to the right of the rectal vault and a soft tissue density in presacral space (arrows).

**Figure 2:**
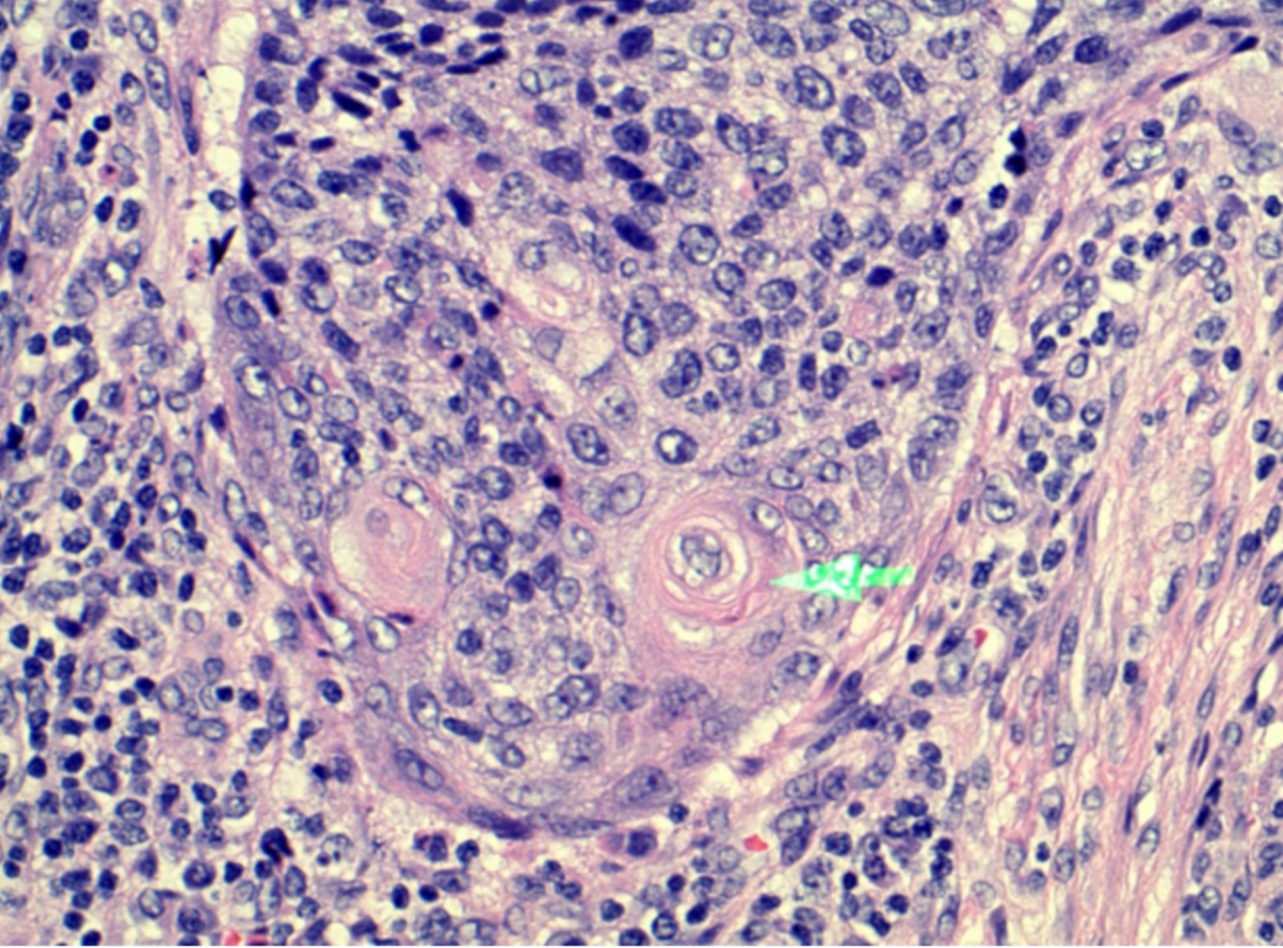
Histological slide showing squamous cell carcinoma of the rectal mucosa.

A 51-year-old woman presented with hematochezia and weight loss. The patient reported worsening of hematochezia over the last two months with 30 pounds of weight loss over the last three to four months. Upon review of the system, the patient stated that she had constant squeezing sacral pain for the past three weeks but denied nausea, vomiting, diarrhea, constipation, loss of appetite, or anal intercourse. The patient denied having a colonoscopy in the past. On physical examination, digital rectal examination revealed sacral tenderness with no appreciation of rectal mass. No signs of lymphadenopathy were noted. Laboratory testing revealed a hemoglobin level of 12.7 g/dL and carcinoembryonic antigen (CEA) of 5.4 ng/mL. Computed tomography (CT) scan of the abdomen and pelvis showed a 17-mm perirectal abscess with thickening of the rectal wall (Figure 1). Soft tissue density was also identified in the pre-sacral space. Examination under anesthesia showed extremely friable rectal mucosa with a 5-cm posterior rectal wall mass with multiple lobulations palpable just distal to the anal verge. This mass extended right up to the anal verge. Histology of the mass revealed poorly differentiated, focally keratinizing SCC (Figure 2). Immunohistochemical stain for high-risk HPV p16 was positive. The patient was discharged with a plan to follow up with the oncology service and colorectal surgery for chemoradiation.

## Discussion

Almost 90% of rectal cancer are adenocarcinoma, and the rest 10% consist of carcinomas, sarcomas, and lymphoid tumors [4]. SCC of the gastrointestinal tract commonly affects the esophagus and anal canal. SCC of the rectum accounts for 0.3% of all histological subtypes of rectal cancers [5]. The first case of SCC of the colon was reported in 1919, but the first case of SCC of the rectum was reported by Raiford in 1933 [6]. Although SCC can be found throughout the colorectum, 93.4% are found in the rectum and 3.4% in the right colon [5]. In the year 2000, a large population-based study by the National Cancer Institute estimated the incidence of rectal SCC to be at 1.9 per million. The estimated incidence of rectal SCC was estimated to be increased at 3.5 per million population in 2007 [7].

Although the etiology and pathogenesis of SCC are unclear, there have been multiple possible hypotheses regarding the pathophysiology of the SCC. These theories propose that chronic inflammatory bowel disease, inflammation due to infection, mucosal injury, squamous cell differentiation by pluripotent stem cells, or radiation exposure can lead to squamous metaplasia [4,8,9]. Pre-existing adenomas or adenocarcinomas and associated HPV infection have been hypothesized to develop into SCC [10,11].

There are many reported cases of HPV causing squamous cell cancers of skin, oral, vaginal, penile, esophageal, and anal canal. The effects of HPV transfection into adenocarcinoma cells were first studied by Kinjo et al. in 2003 where they transfected HPV-16 into cultured colonic adenocarcinoma and lung adenocarcinoma cells. The results of the study showed that squamous metaplasia was most conspicuous in the HPV-transfected colonic cells compared with lung adenocarcinoma cells. Although there are no reported cases of HPV directly causing squamous metaplasia of the rectum, Kinjo et al. reported a clear association between HPV transfection and squamous metaplasia of colonic adenocarcinoma cells [12].

Rectal SCC is a very rare malignancy, as mentioned earlier, but it presents in a similar fashion as adenocarcinoma of the rectum. Patients usually presents with hematochezia, abdominal/rectal pain, and weight loss. Rectal SCC is frequently an extension of anal or gynecological malignancy; therefore, it is important to distinguish distant site metastasis from primary SCC of the rectum. In 1979, Williams et al. [13] proposed the rectal SCC criteria which include the following: (1) exclusion of primary SCC from distant sites; (2) exclusion of a squamous lined fistulous tract; (3) SCC must originate from the rectum and not an extension of SCC of the anus; and (4) histological confirmation of SCC.

Due to rarity of rectal SCC, the optimal treatment is not well established. Historically, SCC of the rectum was treated like rectal adenocarcinoma with surgical resection and in some cases preceded or followed by adjuvant chemotherapy or radiotherapy. However, in the recent decade, rectal SCC is treated in the same manner as anal SCC due to chemoradiotherapy having a promising response in anal SCC. The Nigro protocol has been validated in multiple randomized controlled trials. It has become the accepted standard treatment for anal SCC, and surgery has subsequently become the preferred salvage therapy [14]. Guerra et al. studied all the anal SCC cases reported in the literature from 1933 to 2016. They concluded that overall survival (OS) was 86% for chemoradiotherapy group versus 48% for the conventional treatment group [14]. In addition, Guerra et al. reported the local recurrence and metastatic rates to be 25% versus 10% and 30% versus 13% for chemoradiation and conventional treatment cohorts, respectively [14]. Sturgeon et al. followed 14 patients for 4.5 years with SCC of the rectum who were treated with chemoradiation. Of the 14 patients, 3 had a relapse, of whom 2 of these underwent successful salvage surgery. The remaining 11 patients had no evidence of local or distant relapse. They concluded that five-year OS, disease-free survival, and disease-specific survival rates were 81%, 72%, and 88%, respectively [15]. In 2020, Kommalapati et al. analyzed 3,405 cases of SCC of the rectum from the National Cancer Database between 2004 and 2015. It was found that patients with stages I-III who received only chemoradiation had a better median OS of 108 months when compared with the median OS of 76 months with surgery alone. In stage IV disease, administration of chemoradiation was associated with better OS [1].

## Conclusions

SCC of the rectum is a rare entity of carcinoma. Considering the rising number of cases, further studies are warranted to formulate a tailor-made work-up for SCC of the rectum. Though there are multiple etiologies of the rectal SCC reported in the literature, HPV transfection into adenocarcinoma cells clearly induced squamous metaplasia. We believe that HPV infection in the rectal adenocarcinoma cells likely induced squamous metaplasia in our patient. Further research is needed to identify the utility of screening patients at high risk of HPV infection for rectal SCC.
